# Phase Ib/II randomized, open-label study of doxorubicin and cyclophosphamide with or without low-dose, short-course sunitinib in the pre-operative treatment of breast cancer

**DOI:** 10.18632/oncotarget.11596

**Published:** 2016-08-25

**Authors:** Andrea L.A. Wong, Raghav Sundar, Ting-Ting Wang, Thian-C Ng, Bo Zhang, Sing-Huang Tan, Thomas I.P. Soh, Angela S.L. Pang, Chee-Seng Tan, Samuel G.W. Ow, Lingzhi Wang, Jannet Mogro, Jingshan Ho, Anand D. Jeyasekharan, Yiqing Huang, Choon-Hua Thng, Ching-Wan Chan, Mikael Hartman, Philip Iau, Shaik A. Buhari, Boon-Cher Goh, Soo-Chin Lee

**Affiliations:** ^1^ Department of Haematology-Oncology, National University Cancer Institute, National University Health System, Singapore; ^2^ Haematology Oncology Research Group, National University Cancer Institute, National University Health System, Singapore; ^3^ Cancer Science Institute, National University of Singapore, Singapore; ^4^ Clinical Imaging Research Centre, National University of Singapore, Singapore; ^5^ Department of Pharmacology, Yong Loo Lin School of Medicine, National University Health System, Singapore; ^6^ Department of Diagnostic Imaging, National Cancer Centre, Singapore; ^7^ Department of Surgical Oncology, National University Cancer Institute, National University Health System, Singapore

**Keywords:** vascular normalization, anti-angiogenic therapy, sunitinib, neoadjuvant chemotherapy, breast cancer

## Abstract

**Background:**

Prolonged anti-angiogenic therapy destroys tumor vasculature, whereas vascular-normalizing doses may enhance intra-tumoral drug delivery. We hypothesize that low-dose, short-course sunitinib normalizes vasculature, enhancing chemotherapy efficacy.

**Patients and Methods:**

In phase Ib, treatment-naïve breast cancer patients received four cycles of pre-operative doxorubicin/cyclophosphamide, with sunitinib before each cycle. The optimal dose of sunitinib leading to tumor vessel normalization on immunohistochemistry was identified. In phase II, subjects were randomized to chemotherapy alone or chemotherapy plus sunitinib at the recommended phase II dose (RP2D). Primary endpoint was pathological complete response (pCR) rate. Tumor and functional imaging biomarkers were evaluated serially.

**Results:**

In phase Ib (*n*=9), sunitinib 12.5 mg daily for 7 days before each chemotherapy was established as RP2D. In phase II, patients receiving chemotherapy plus sunitinib (*n*=24) had similar pCR rates (5.0% *versus* 4.3%, *p*=1.00), but a higher incidence of chemotherapy dose delays (33.3% *versus* 8.7%, *p*=0.04), compared to those receiving chemotherapy alone (*n*=25). The addition of sunitinib to chemotherapy significantly increased vascular normalization index (VNI) and decreased lymphatic vessel density (D2-40) on immunohistochemistry [VNI:25.50±27.94% *versus* 49.29±31.84%, *p*=0.034; D2-40:3.29±2.70 *versus* 1.29±1.54, *p*=0.014, baseline *versus* post-cycle 1], and improved perfusion on DCE-MRI (*K^trans^*:12.6±9.6 mL/100 g/min *versus* 16.3±10.7 mL/100 g/min, baseline *versus* post-cycle 1, *p*=0.015). Conversely, immunohistochemical and DCE-MRI parameters were not significantly altered by chemotherapy alone.

**Conclusion:**

Low-dose, short-course sunitinib prior to anthracycline-based chemotherapy in breast cancer patients did not improve pCR and increased chemotherapy dose delays. However, the addition of sunitinib induced compelling pharmacodynamic evidence of vascular normalization. Further studies with alternative cytotoxic regimens should be explored.

## INTRODUCTION

Pathological complete response (pCR) rates remain modest at 5-30% in unselected breast cancer populations receiving standard-of-care anthracycline and taxane-based regimens pre-operatively [1, 2]. This therapeutic ceiling underscores the need to increase the efficacy of individual cytotoxic agents or to develop novel therapeutic combinations.

Multiple studies have generally failed to demonstrate significant benefit in adding anti-angiogenic agents to chemotherapy in breast cancer patients [3–6]. These somewhat disappointing results may be contributed by lack of an optimal dose schedule and the paucity of clinically-relevant biomarkers [4]. Tumor microvessels are structurally and functionally immature, impeding intra-tumoral delivery of cytotoxics. In a seminal publication, Rakesh Jain proposed the “vascular normalization” hypothesis, where judicious anti-angiogenic therapy normalizes tumor vasculature and enhances cytotoxic delivery. However, high or prolonged dosing subsequently causes excessive pruning and blood vessel destruction, impeding intra-tumoral chemotherapy delivery when administered concurrently [7, 8].

We hypothesized that the administration of a dose-attenuated anti-angiogenic agent prior to chemotherapy would normalize tumor vasculature and enhance chemotherapy delivery. We conducted a phase Ib trial to define the optimal dose of sunitinib required to normalize tumor vasculature on immunohistochemistry (IHC), followed by a randomized phase II study on clinical and pharmacodynamic effects of low-dose, short-course sunitinib prior to standard anthracycline-based pre-operative chemotherapy in treatment-naïve locally advanced or metastatic breast cancer patients.

## RESULTS

This was a single-centre study conducted between April 2011 and November 2014. Nine patients were enrolled in phase Ib, whilst 49 patients participated in the phase II randomized component of the study.

### Phase Ib: dose-escalation

**Figure 1:**
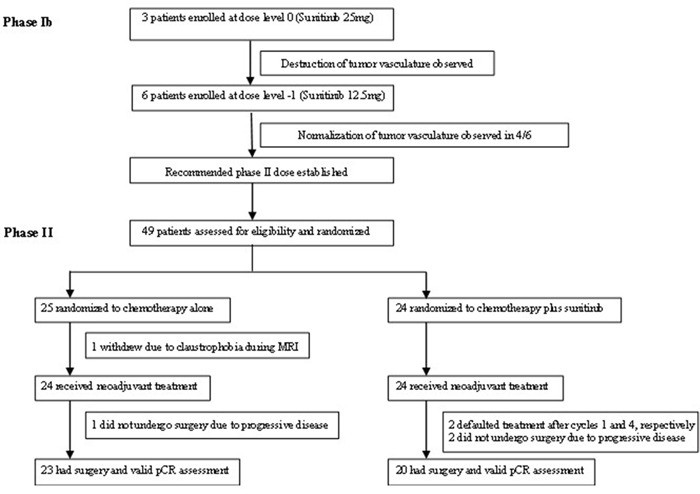
CONSORT diagram: trial profile

Patients’ baseline characteristics are listed in Table 1 and dose-escalation guidelines are shown in Supplementary Figure S2. Three patients were enrolled into the dose level 0 cohort (PO sunitinib at 25 mg daily for 7 days), but only one patient had a post-sunitinib biopsy specimen which was adequate for IHC analysis. This demonstrated tumor vessel destruction, with a 93% decrease in vascular normalization index (VNI) from baseline. Furthermore, all three patients in this cohort developed febrile neutropenia (treatment-related toxicities; see below). Therefore, we moved to the next lower dose level −1 (PO sunitinib at 12.5 mg daily for 7 days) in accordance with the dose-escalation protocol. Six patients were enrolled at this dose level. Post-sunitinib biopsy demonstrated tumor vessel normalization in four out of six patients (mean VNI increase from baseline: 106.8±123.4%) (Figure 1). This was therefore established as the recommended phase II dose.

**Table 1:** Patient baseline characteristics

Characteristic	Number (Percentage)			*p* value*
	Phase Ib (*n*=9)	Phase II AC (*n*=25)	Phase II AC + Sunitinib (*n*=24)	
**Age (years)**				
Median	50	48	49	
Range	(36-71)	(33-70)	(34-70)	
**T stage of primary tumour**				
T1-2	2 (22.2)	8 (32.0)	9 (37.5)	0.69
T3-4	7 (77.8)	17 (68.0)	15 (62.5)	
**Baseline tumour size (cm)**				
Mean	10.7	6.5	6.4	0.93
Standard deviation	6.3	4.4	2.9	
**Clinical nodal status**				
N0	5 (55.6)	11 (55.0)	4 (20.0)	***0.02***
N1-3	4 (44.4)	9 (45.0)	16 (80.0)	
**Hormone receptor status**				
ER or PR positive	7 (77.8)	22 (88.0)	16 (66.7)	0.10
ER and PR negative	2 (22.2)	3 (12.0)	8 (33.3)	
**HER2 status**				
HER2 positive	3 (33.3)	1 (4.0)	3 (12.5)	0.35
HER2 negative	6 (66.7)	24 (96.0)	21 (87.5)	
**Tumour grade**				
Grade 1-2	3 (33.3)	11 (45.8)	8 (34.8)	0.44
Grade 3	6 (66.7)	13 (54.2)	15 (65.2)	
**Metastatic disease**				
Present	3 (33.3)	1 (4.0)	3 (12.5)	0.29
Absent	6 (66.7)	24 (96.0)	21 (87.5)	
**Race**				
Chinese	6 (66.7)	20 (80.0)	15 (62.5)	0.32
Malay	2 (22.2)	3 (12.0)	7 (29.2)	
Others	1 (11.1)	2 (8.0)	2 (8.3)	

* Chi-square test or Students’ *t*-test between the “AC” and “AC + Sunitinib” arms of the phase II study; bold, italicized *p* values denote statistically significant results

### Phase Ib: toxicities and clinical outcomes

Treatment-related toxicities are summarized (Table 2). In the 25 mg cohort (*n* =3), febrile neutropenia occurred in all three patients, while dose delays and reductions occurred in two of the three patients. The mean relative dose intensities (RDI) of doxorubicin/cyclophosphamide and sunitinib were 90.3±8.7% and 83.3±14.4%, respectively. Two patients achieved objective clinical response after four cycles of chemotherapy but none achieved pCR.

**Table 2:** Hematologic toxicities (all cycles), dose delays, dose reductions and relative dose intensity according to dose level (phase Ib) and treatment arm (phase II)

Outcome	Number (Percentage)				*p* value*
	Phase Ib 25 mg (*n*=3)	Phase Ib 12.5 mg (*n*=6)	Phase II AC (*n*=24)	Phase II AC + Sunitinib (*n*=24)	
**Grade 3 and 4 leukopenia**	3 (100)	6 (100)	24 (100)	2 (90.9)	0.22
**Grade 4 leukopenia**	2 (66.7)	3 (50)	9 (37.5)	11 (50)	0.39
**Grade 3 and 4 neutropenia**	3 (100)	6 (100)	24 (100)	21 (95.5)	0.48
**Grade 4 neutropenia**	3 (100)	6 (100)	23 (95.8)	19 (86.4)	0.34
**Grade 3 and 4 anemia**	0	1 (16.7)	3 (12.5)	1 (4.5)	0.61
**Febrile neutropenia**	3 (100)	1 (16.7)	7 (29.2)	8 (33.3)	0.76
**Dose delays**	2 (66.7)	1 (16.7)	2 (8.7)	8 (33.3)	***0.04***
**Dose reductions**	2 (66.7)	1 (16.7)	6 (25.0)	8 (33.3)	0.53
**Doxorubicin mean RDI (± SD)^a^**	90.3 ± 8.7%	96.3 ± 9.0%	96.7 ± 6.4%	92.2 ± 11.0%	0.09
**Cyclophosphamide mean RDI (± SD)**	90.3 ± 8.7%	96.3 ± 9.0%	96.7 ± 6.4%	92.2 ± 11.0%	0.09
**Sunitinib mean RDI (± SD)**	83.3 ± 14.4%	95.8 ± 10.2%	-	92.0 ± 15.0%	-

a **RDI (± SD)**: Relative dose intensity ± standard deviation;

* Chi-square test or Students’ *t*-test between the “AC” and “AC + Sunitinib” arms of the phase II study; bold, italicized *p* values denote statistically significant results.

In the 12.5 mg cohort (*n* =6), the rates of febrile neutropenia, dose delays and dose reductions were 16.7% each. The mean RDI of doxorubicin/cyclophosphamide and sunitinib were 96.3±9.0% and 95.8±10.2%, respectively. All six patients achieved objective clinical response after four cycles of chemotherapy, and of the four patients who underwent surgery, one achieved pCR (25%).

### Randomized phase II study

Twenty-five subjects were assigned to receive chemotherapy alone, whilst 24 were assigned to receive chemotherapy plus sunitinib (Figure 1). One patient randomized to chemotherapy alone withdrew consent prior to commencing treatment. Patients’ baseline characteristics are described in Table 1. The groups were well-balanced apart from a higher percentage of clinically node-positive patients receiving chemotherapy plus sunitinib compared to chemotherapy alone (80% *versus* 45%, *p*=0.02). The majority had large primary tumors at presentation; mean primary breast tumor size was 6.44±3.67 cm, 65.3% had clinical T3/T4 disease and 8.2% had metastatic disease.

### Clinical and pathological outcomes

Forty-three patients underwent surgery, all of whom had valid pathological response assessment (Figure 1). There were no statistically significant differences in pCR rates (5.0% *versus* 4.3%, *p*=1.00), histologically-negative lymph nodes at surgery (35.0% *versus* 34.7%, *p*=0.89), objective clinical response rates after one and four cycles of chemotherapy (60.9% *versus* 34.8%, *p*=0.08; 90.9% *versus* 91.3%, *p*=1.00, respectively), rates of axillary lymph node downstaging (11.8% *versus* 0%, *p*=0.22), and breast conserving surgery in patients without metastatic disease (20.0% *versus* 9.1%, *p*=0.31), in patients receiving chemotherapy plus sunitinib versus chemotherapy alone (Table 3).

**Table 3:** Clinical and pathological outcomes according to dose level (phase Ib) and treatment arm (phase II)

Outcome	Number (Percentage)				*p* value*
	Phase Ib 25 mg (*n*=3)	Phase Ib 12.5 mg (*n*=6)	Phase II AC (*n*=24)	Phase II AC + Sunitinib (*n*=23)	
**Objective response rates^a^**					
Clinical response post Cycle 1			8 (34.8)	14 (60.9)	0.08
Clinical response post Cycle 4	2 (66.7)	6 (100)	21 (91.3)	20 (90.9)	1.00
**Good histologic response^b^**	0	3 (60)	6 (46.2)	9 (60)	0.71
**No. of patients who underwent surgery**	**(*n* = 3)**	**(*n* = 4)**	**(*n* = 23)**	**(*n* = 20)**	
**Pathologic complete response (pCR)**	0	1 (25)	1 (4.3)	1 (5.0)	1.00
**Histologically negative lymph nodes at the time of surgery (ypN0)**	1 (33.3)	2 (50)	8 (34.7)	7 (35.0)	0.89
**Axillary lymph node downstaging^c^**	0	1 (25)	0	2 (11.8)	0.22
**Breast conserving surgery^d^**	1 (33.3)	2 (50)	2 (8.7)	4 (20.0)	0.39

a Sum of complete and partial response according to RECIST v1.1;

b Grade 3-5 histologic response after 1 cycle of chemotherapy (“25 mg dose level”, *n*=1; “12.5 mg dose level”, *n*=5; “AC arm”, *n*=13; “AC + Sunitinib arm”, *n*=15);

c Axillary lymph nodes clinically involved at diagnosis, histologically negative at the time of surgery (AC arm, *n*=19, AC + Sunitinib arm, *n*=17);

d Amongst patients without metastatic disease in the phase II study, rates of breast conserving surgery were 9.1% *versus* 20.0%, *p*=0.31 (AC arm, *n*=22, AC + Sunitinib arm, *n*=20);

* Chi-square test between the “AC” and “AC + Sunitinib” arms of the phase II study.

The mean length of follow-up was 34.1 months. The mean relapse-free survival was 37.0±4.7 months *versus* 37.1±4.0 months, *p*=0.70 (Supplementary Figure S1a), whilst mean overall survival was 47.6±4.0 months versus 49.7±3.3 months, p=0.74 (Supplementary Figure S1b), in patients receiving chemotherapy plus sunitinib compared to those receiving chemotherapy alone.

### Safety

Grade 3 and above non-hematologic toxicities (stomatitis, non-neutropenic fever and lethargy) occurred in three patients (12.5%) in the chemotherapy plus sunitinib arm, whereas none were observed with chemotherapy alone (*p*=0.23). Hematologic toxicities were frequent but not significantly different between both treatment arms (Table 2). Patients receiving sunitinib experienced significantly more dose delays (33.3% *versus* 8.7%, *p*=0.04), and had marginally lower mean RDI of doxorubicin/cyclophosphamide (92.2%±11.0% *versus* 96.7±6.4%, *p*=0.09), compared to those receiving chemotherapy alone (Table 2), although the difference did not reach statistical significance.

### Pharmacodynamic assessments

#### Immunohistochemistry

Post-cycle 1 and post-cycle 4 tumor biopsies were available for 18 and 13 patients, respectively, on the chemotherapy alone arm, as well as 16 and 11 patients, respectively, on the chemotherapy plus sunitinib arm. The rates of good histological response after 1 cycle of chemotherapy were similar in both arms (60.0% *versus* 46.2%, chemotherapy plus sunitinib *versus* chemotherapy alone, *p*=0.71) [Table 3].

**Figure 2:**
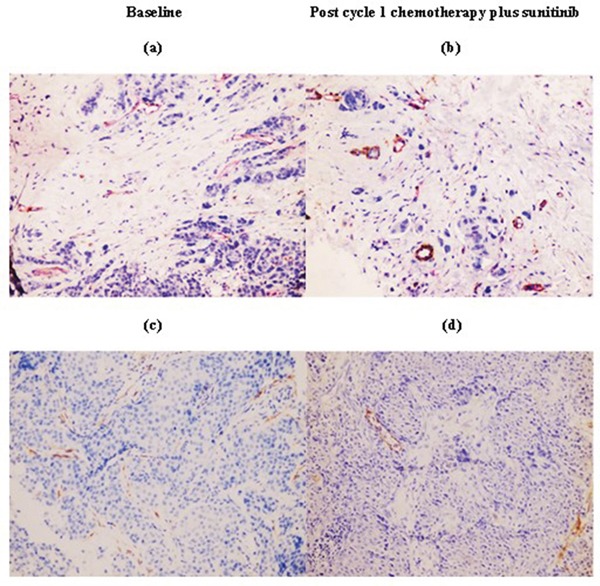
Immunohistochemistry staining was performed at baseline and after one cycle of chemotherapy plus sunitinib in a representative patient (200x magnification) Compared with immunoreactivity levels in baseline tumors **a.** and **c.**, chemotherapy plus sunitinib led to increased vascular normalization index [ratio of α-SMA(brown)/CD31(red)] **b.** and decreased lymphatic vessel density as determined by D2-40 **d.**

Serial changes in tumor microvessel and lymphatic density on IHC were observed in the chemotherapy plus sunitinib but not the chemotherapy alone arm (Table 4). In the chemotherapy plus sunitinib arm, mean numbers of α-SMA-positive cells (indicative of mature, normalized blood vessels) and VNI were significantly increased after one cycle of treatment compared to baseline (5.14±4.15 *versus* 2.79±2.89, *p*=0.032; 49.29±31.84% *versus* 25.50±27.94%, *p*=0.034, respectively). The mean increase in VNI remained significant after four cycles of treatment (60.86±20.05% *versus* 25.50±27.94%, *p*=0.045). Moreover, there was a significant decline in mean number of D2-40-positive cells after one cycle of treatment compared to baseline (1.29±1.54 *versus* 3.29±2.70, *p*=0.014), although the difference was not statistically significant after four cycles (*p*=0.085) [Table 4]. Figure 2 is a representative tumor sample in which an increase in VNI and decrease in lymphatic vessel density was observed after one cycle of chemotherapy plus sunitinib. Conversely, there were no significant changes in IHC parameters in the chemotherapy alone arm (Table 4).

**Table 4:** Pharmacodynamic biomarker parameters according to phase II treatment arm: Paired sample *t*-tests of mean post-cycle 1 DCE-MRI parameters as well as post-cycle 1 and post-cycle 4 IHC parameters compared to baseline

	AC alone					AC+Sunitinib				
**IHC parameters**										
	**Baseline (Mean ± SD)**	**Post-cycle 1 (Mean ± SD), *n*=18**	*p* value	**Post-cycle 4 (Mean ± SD), *n*=13**	*p* value	**Baseline (Mean ± SD)**	**Post-cycle 1 (Mean ± SD), *n*=16**	*p* value	**Post-cycle 4 (Mean ± SD), *n*=11**	*p* value
No.of SMA-positive cells (x200)	5.50 ± 9.10	7.00 ± 5.26	0.496	6.22 ± 3.93	0.920	2.79 ± 2.89	5.14 ± 4.15	***0.032***	8.14 ± 8.78	0.152
No. of CD31-positive cells (x200)	17.07 ± 11.32	20.71 ± 11.49	0.374	16.22 ± 9.24	0.504	9.77 ± 7.62	9.62 ± 6.05	0.929	14.00 ± 14.19	0.380
Vascular normalization index (%)^a^	24.29 ± 23.67	34.50 ± 17.68	0.063	43.11 ± 24.81	0.160	25.50 ± 27.94	49.29 ± 31.84	***0.034***	60.86 ± 20.05	***0.045***
No. of D240-positive cells (x200)	0.84 ± 1.50	1.42 ± 2.24	0.225	1.08 ± 1.98	0.213	3.29 ± 2.70	1.29 ± 1.54	***0.014***	1.18 ± 1.47	0.085
**DCE-MRI parameters**										
	**Baseline (Mean ± SD)**	**Post-cycle 1 (Mean ± SD), *n*=17**	*p* value			**Baseline (Mean ± SD)**	**Post-cycle 1 (Mean ± SD), *n*=20**	*p* value		
*K^trans^* (mL/100 g/min)	17.1 ± 9.3	15.7 ± 8.4	0.572			12.6 ± 9.6	16.3 ± 10.7	***0.015***		
V_p_ (%)	9.4 ± 3.9	9.4 ± 4.6	0.990			7.0 ± 4.1	9.5 ± 5.4	***0.031***		
V_e_ (%)	23.4 ± 7.7	27.5 ± 14.5	0.201			19.2 ± 7.4	27.8 ± 12.4	***0.006***		
*PS* (mL/100 g/min)	20.6 ± 12.2	18.9 ± 11.0	0.610			15.2 ± 12.6	20.5 ± 17.2	***0.025***		
*F* (mL/100 g/min)	113.8 ± 46.2	102.5 ± 37.3	0.472			89.5 ± 58.6	106.2 ± 59.0	0.167		
Tumor volume (cm^3^)	43.7± 55.2	38.5± 56.4	***0.016***			32.3± 29.7	22.4± 25.3	***0.003***		

a Vascular normalization index = Percentage of CD31-positive cells which co-express SMA;

*K^trans^*= Volume transfer constant between plasma and extracellular extravascular space; V_p_=Fractional plasma volume; V_e_= Fractional extravascular, extracellular volume; *PS*=Vascular permeability; *F*= Perfusion; bold, italicized *p* values denote statistically significant results

#### Dynamic contrast enhanced–MRI

Seventeen patients receiving chemotherapy alone and 20 patients receiving chemotherapy plus sunitinib completed all scheduled DCE-MRI scans. Chemotherapy plus sunitinib but not chemotherapy alone induced functional changes in tumor vasculature on serial imaging (Table 4). The mean values of all functional imaging parameters, except for perfusion (*F*), were significantly increased after one cycle of chemotherapy plus sunitinib treatment compared to baseline: *K^trans^* (16.3±10.7 mL/100 g/min *versus* 12.6±9.6 mL/100 g/min, *p*=0.015), V_p_ (9.5±5.4% *versus* 7.0±4.1%, *p*=0.031), V_e_ (27.8±12.4% *versus* 19.2±7.4%, *p*=0.006), *PS* (20.5±17.2 mL/100 g/min *versus* 15.2±12.6 mL/100 g/min, *p*=0.025), *F* (106.2±59.0 mL/100 g/min *versus* 89.5±58.6 mL/100 g/min, *p*=0.167) [Table 4]. Conversely, there were no significant changes in DCE-MRI parameters in the chemotherapy alone arm (Table 4).

In subset analysis according to histological response, the mean increase in *K^trans^* after cycle 1 was 49.2±49.2% in patients with good histological response (*n*=8), compared to a mean decline of 10.6±32.5% in patients with poor histological response (*n*=5), (*p*=0.036). There were no other significant correlations between DCE-MRI and IHC parameters.

## DISCUSSION

There is extensive preclinical data supporting the “vascular normalization” hypothesis. Furthermore, a correlation between vascular normalization and the clinical activity of cediranib, an oral anti-vascular endothelial growth factor receptor agent, has been described in glioblastoma multiforme patients [9, 10]. However, few clinical studies have attempted to define the optimal dose of anti-angiogenic therapy required to induce vascular normalization, and none have investigated the strategy of delivering short-course anti-angiogenic therapy during the presumed vascular normalization window to enhance chemotherapy efficacy.

We therefore evaluated this novel therapeutic strategy by adding low-dose, short-course sunitinib to pre-operative anthracycline-based chemotherapy in breast cancer patients. Sunitinib 12.5 mg daily for 7 days prior to chemotherapy was established as the recommended phase II dose based on immunohistochemical evidence of vascular normalization in our dose-finding phase Ib study. While the addition of sunitinib to chemotherapy did not improve our primary endpoint of pathological complete response rates in our randomized phase II study, we observed definite pharmacodynamic signals that support its biological activity. Most notably, low-dose short-course sunitinib induced vascular normalization on immunohistochemistry, evidenced by a statistically significant serial increase in the vascular normalization index in the chemotherapy plus sunitinib but not the chemotherapy alone arm. This was accompanied by improved vascular perfusion within the tumor microenvironment on functional imaging in patients receiving sunitinib.

The optimal timing of short-course anti-angiogenic therapy in relation to chemotherapy delivery is of critical importance in the further development of this therapeutic strategy. Although the current literature lacks clarity on the precise window of tumor vascular normalization, cedirinab induced vascular normalization changes as early as one day after initiation of treatment, whereas reversal of these changes took place 4-6 weeks later [9, 10]. Similarly, we observed vascular normalization changes within 7 days of sunitinib initiation and found that the maximal histologic effect occurred by the end of the first cycle of chemotherapy, or 4 weeks after initiation of sunitinib. This was evidenced by a statistically significant, near-doubling of the vascular normalization index after the first cycle of chemotherapy compared to baseline. Although the effect persisted with additional cycles of treatment, further increase in vascular normalization index between cycles 1 and 4 of chemotherapy was modest. Taken together, we hypothesize that the maximal effect of the vascular normalization strategy occurs within 4-6 weeks of induction anti-angiogenic therapy, making this approach particularly relevant to shorter treatment regimens, such as concurrent chemoradiotherapy. However, this needs to be confirmed through the optimal scheduling of pharmacodynamic assessments in future studies.

Interestingly, sunitinib also led to lymphatic vessel normalization, demonstrated by a significant decline in lymphatic vessel density (D2-40 positivity) after 1 cycle of chemotherapy. Studies have proposed that lymphangiogenesis has an active role in cancer metastasis, and the presence of D2-40-positive lymphovascular invasion has an adverse effect on breast cancer survival [11, 12]. While these preliminary findings support an inhibitory effect of short-course anti-angiogenic agents on tumor lymphangiogenesis, further *in-vitro* and *in-vivo* confirmation is needed. DCE-MRI functional imaging has been used to evaluate effects of anti-angiogenic agents in multiple clinical trials. *K^trans^* describes trans-endothelial diffusion of contrast media into the extravascular space, and is the most widely-accepted pharmacodynamic biomarker of anti-angiogenesis [13]. In the majority of studies, a decline in *K^trans^* has been found to correlate with drug exposure and tumor response, reflecting the destructive effect of high-dose anti-angiogenic therapy on tumor vasculature [14, 15]. However, an increase in *K^trans^* has been reported in studies where anti-angiogenic treatment has normalized tumor vasculature [16, 17]. In our study, the significant rise in *K^trans^* observed as early as cycle 1 in patients receiving sunitinib provided functional proof of improved vascular perfusion, which in turn correlated positively with histological response. Far fewer studies have proven the utility of other DCE-MRI parameters (V_p_, V_e_, *PS* and *F*) as functional imaging biomarkers. In a phase I study of the VEGFR TKI, ABT-869, we demonstrated a correlation between drug exposure and a decline in all of these parameters, indicating tumor vessel destruction [15]. To what extent the increase in these parameters observed in our current study reflects tumor vessel normalization requires further validation.

Despite the clear evidence of vascular normalization on immunohistochemistry and perfusion imaging, we were unable to demonstrate the clinical benefit of adding low-dose, short-course sunitinib to anthracycline-based chemotherapy in breast cancer patients. Signals of efficacy were observed in patients receiving sunitinib, in particular, the near doubling of objective clinical response rates with corresponding improvements in histological response after one cycle of chemotherapy. We acknowledge that neither of these is an accepted response parameter and the lack of statistical significance precludes firm conclusions. However, we consider them to be noteworthy because in contrast to all subsequent clinical and histopathological outcome measures, cycle 1 outcomes are not biased by chemotherapy dose delays. We have identified several factors which may have contributed to our negative clinical and pathological endpoints. Firstly, we observed that the addition of sunitinib was associated with significantly more chemotherapy dose delays in our phase II study, leading us to hypothesize that the initiation of sunitinib at the hematologic nadir of each chemotherapy cycle delayed the recovery of neutrophil counts. Use of this strategy with alternative chemotherapy regimens or with primary prophylactic granulocyte colony-stimulating factor support is a possible consideration for future studies. Other limitations of our study include the small sample size, the trial being conducted only in a single centre, and the lack of stratification according to breast cancer subtype.

Our decision to de-escalate the dose of sunitinib from 25 mg to 12.5 mg in the phase Ib study was based on just one patient's post-sunitinib tumor biopsy because the post-sunitinib biopsy samples of the other two patients enrolled in the same cohort were inadequate for analysis. Conclusions may have been more robust if we had enrolled more patients in each dose cohort or replaced patients with inadequate biopsy samples. However, this particular biopsy clearly demonstrated tumor vessel destruction, and according to our dose-escalation guidelines, this warranted enrolling subsequent patients at the next lower dose level. Furthermore, all three patients at this dose level developed febrile neutropenia, suggesting that the 25 mg dose level was not tolerable and enrolling further patients into the cohort would not have been justifiable. The next lower dose level of 12.5 mg met the histological criteria for evidence of vascular normalization in four out of six patients, and was also found to be tolerable with standard doses of chemotherapy in our dose-finding study. Since our goal was to find the lowest dose of sunitinib that could normalize tumor vasculature, we established sunitinib 12.5 mg for 7 days as the recommended phase II dose. Whether an even shorter duration of sunitinib treatment would achieve similar pharmacodynamic effects remains an interesting question, which is best addressed through a separate study.

In keeping with other studies evaluating the impact of anti-angiogenic therapy on tumor vessel normalization, we used a combination of tumor immunohistochemistry and MRI perfusion imaging to define tumor vasculature normalization [9, 10]. However, we do acknowledge that these methods fail to quantify the parameter of genuine interest – intra-tumoral cytotoxic drug concentration. While this has been explored in animal models, there are presently no robust or clinically validated assays available for the determination of intra-tumoral drug pharmacokinetics in human subjects [18]. Immunohistochemical evaluation of tumor angiogenesis is currently accepted as the gold standard [20], which we used in combination with functional imaging to measure tumor blood flow in this study to guide dose-escalation decisions.

## CONCLUSIONS

Addition of low-dose, short-course sunitinib to standard anthracycline-based neoadjuvant chemotherapy in breast cancer patients demonstrated compelling pharmacodynamic effects on immunohistochemistry and functional imaging, supporting the “vascular normalization” hypothesis. However, sunitinib led to more dose delays when combined with anthracycline-based chemotherapy. We propose future studies evaluating this treatment strategy with primary prophylactic granulocyte colony-stimulating factor support or alternative cytotoxic regimens.

## MATERIALS AND METHODS

### Study population

Eligibility criteria included female patients aged ≥18 years with histologically confirmed treatment-naïve breast cancer planned for surgery, measurable primary tumor ≥2.0 cm, Karnofsky performance score ≥70, absolute neutrophil count ≥1.5×10^9^/l, platelets ≥100×10^9^/l, serum total bilirubin ≤1.5x upper limit of normal (ULN), alanine aminotransferase and aspartate aminotransferase ≤2.5xULN, serum creatinine ≤1.5xULN and left ventricular ejection fraction ≥50%. Significant exclusions were central nervous system metastases and clinically detectable second malignancies.

### Treatment plan

In phase Ib, subjects received four cycles of pre-operative chemotherapy (3-weekly intravenous doxorubicin 60 mg/m^2^ and cyclophosphamide 600 mg/m^2^) with oral sunitinib prior to each cycle. We aimed to determine the lowest dose of sunitinib required to achieve tumor vessel normalization on the post-sunitinib biopsy obtained on completion of induction sunitinib, prior to the initiation of cycle 1 chemotherapy. The starting dose was PO sunitinib 25 mg daily for 1 week prior to each cycle of chemotherapy. Dose escalation/de-escalation was performed in cohorts of three, with expansion to six subjects and enrollment of sequential cohorts according to dose-escalation guidelines (Supplementary Figure S2). The recommended phase II dose was defined as the dose which resulted in tumor vessel normalization in 3/3 or ≥4/6 patients of a cohort. Criteria for tumor vessel normalization and destruction are defined in the section *Pharmacodynamic biomarkers*.

In the open-label phase II study, patients were randomized 1:1 to either four cycles of doxorubicin/cyclophosphamide at the above-mentioned doses, or the same chemotherapy plus sunitinib at the recommended phase II dose. The study schema is shown in Supplementary Figure S3. Primary prophylactic colony stimulating factor was disallowed. Primary endpoint was pCR rate, defined as the absence of all invasive cancer in the breast and axillary lymph nodes at the time of surgery [19]. Secondary endpoints were i) objective clinical response rates (complete plus partial) using RECIST v1.0 criteria, ii) axillary lymph node down-staging (conversion of clinically-involved lymph nodes to histologically-negative nodes at the time of surgery), iii) rates of breast conserving surgery, iv) safety and tolerability, and v) correlation of pharmacodynamic biomarkers [IHC and dynamic contrast enhanced (DCE)-MRI] with clinical outcomes. Uni-dimensional tumor measurements for clinical response assessment were obtained after each cycle of chemotherapy; hematologic parameters were obtained at the start and nadir of each chemotherapy cycle. Patients were evaluated for surgery upon completion of four cycles of chemotherapy or at the time of clinical tumor progression, whichever occurred first. Post-operative therapy was in accordance to institutional guidelines. Relapse and survival follow-up was performed at 3–6 monthly intervals. All participants provided written informed consent and the institutional ethics committee approved the study protocol (ClinicalTrials.gov NCT01176799).

### Pharmacodynamic biomarkers

#### Immunohistochemistry (Phase Ib and II)

Serial tumor core biopsies were obtained at baseline, ~3 weeks after cycle 1 chemotherapy, and upon completion of four cycles of chemotherapy, or at study withdrawal. In patients receiving sunitinib, an additional biopsy was obtained upon completion of induction sunitinib, prior to the initiation of cycle 1 chemotherapy. Biopsies were obtained from primary breast tumors, fixed in formalin and subsequently embedded in paraffin (Supplementary Methods).

Endothelial-specific anti-CD31 antibody was used to detect total tumor microvessel density (MVD), whereas pericyte-specific anti-α-SMA antibody was used to identify mature, normalized blood vessels. The numbers of CD31-positive and α-SMA-positive vessels in the tumor area were counted at 200x magnification. Vascular normalization index (VNI) was defined as the percentage of CD31-positive vessels co-expressing α-SMA, representing the percentage of normalized tumor blood vessels in relation to total MVD [20]. In our phase Ib study, tumor vessel normalization was defined as ≥10% increase in VNI from baseline; tumor vessel destruction was defined as ≥10% decrease in VNI from baseline. D2-40 antibody was used to detect lymphatic vessel density; numbers of lymphatic vessels were counted at 200x magnification. The VNI, MVD and lymphatic vessel density scores were averaged across sections [21]. Degree of histological tumor response was graded on a five-point scale; good histological response was defined as a score of ≥3 [22].

#### Vascular parameters and diffusivity MRI methods (Phase II)

Using a3T whole-body scanner (Magnetom Trio Tim, Siemens AG), patients underwent DCE-MRI scans at baseline and after one cycle of chemotherapy. Patients receiving sunitinib had an additional scan after induction sunitinib, prior to the initiation of cycle 1 chemotherapy. Using the Distributed Parameter (DP) model, 3D quantitative estimate vascular parameters including volume transfer constant between plasma and extracellular extravascular space (*K^trans^*), fractional plasma volume (V_p_), fractional extravascular, extracellular volume (V_e_), vascular permeability (*PS*) and perfusion (*F*), were acquired from DCE-MRI images (Supplementary Methods). This model was selected because it permits separate estimation of perfusion and permeability [23].

### Statistical analysis

Statistics for phase Ib were descriptive. In phase II, a sample size of 42 patients (21 in each arm) was required to provide 90% power to detect an absolute difference in pCR rates of 15% (10% with chemotherapy alone; 25% with chemotherapy plus sunitinib), based on the selection theory approach described by Simon et al [24]. A target sample size of 50 was planned, taking into account an estimated 20% attrition rate for the primary endpoint of pCR. Chi-square test and *t*-test, or corresponding non-parametric tests, were used in correlative analyses of clinical and biomarker variables. Kaplan-Meier methods and log-rank tests were used to assess relapse-free and overall survival. Correlations between treatment arm and serial changes in biomarker parameters were evaluated using paired student's *t*-test. Statistical calculations were computed using SPSS Version 13.0 (SPSS Inc., Chicago); *p*<0.05 was considered statistically significant.

## SUPPLEMENTARY FIGURES AND TABLES


