# Supplementary material for: Phase Ib/II randomized, open-label study of doxorubicin and cyclophosphamide with or without low-dose, short-course sunitinib in the pre-operative treatment of breast cancer
Source: Oncotarget. 2016 Aug 25;7(39):64089–99. doi: 10.18632/oncotarget.11596 (PMC5325427; doi:10.18632/oncotarget.11596)
Supplement: Supplementary file 1 [file oncotarget-07-64089-s001.pdf]

Phase Ib/II randomized, open-label study of doxorubicin and cyclophosphamide with or without low-dose, short-course sunitinib in the pre-operative treatment of breast cancer

SUPPLEMENTARY DATA

Immunohistochemical analysis

As described previously [1], consecutive sections of 4µm thickness were cut and placed on polylysine-coated slides for IHC analysis. All IHC analyses for D2-40 (single-staining), CD31 and α-SMA (double staining) were performed with a BondMaxAutostainer (Leica, Milton Keynes, UK) according to the manufacturer's instructions in a single reference laboratory by blinded breast pathologist (WT). For double staining, CD31 and α-SMA antibodies were incubated sequentially, followed by visualization with alkaline phosphatase-based red and peroxidase-based diaminobenzidine polymer detection systems, respectively. Antibodies, dilutions and suppliers were as follows: D2-40 (1:200), CD31 (1:1500), α-SMA, (1:1500) from DAKO (Denmark).

Vascular parameters and diffusivity MRI methods

Serial normal T_1-weighted and T_2-weighted images were acquired to locate the tumor lesions in the breast(s)-weighted image was acquired by T_1-weighted 3D Spoil Pulsed Gradient Recalled (SPGR) sequence with TR/TE = 140/1.15 ms. The T2-weighted image was acquired by T2-weighted turbo spin echo (TSE) with fat saturation with TR/TE = 2000/70 ms. DCE-MRI data were acquired by a 3D T1-weighted SPGR sequence with a 15 degree flip angle. TR and TE were 5 ms and 2.3 ms, respectively. A total of 2000 images (200 dynamics repeat, 10 slices) were acquired over a period of 7 minutes. During the DCE-MRI scan, patients were asked at the start of the Gd-DTPA injection to hold their breath for as long as they could and then to either hold their breath periodically or breathe in a shallow fashion, depending on their physical condition. In each follow-up study, the 3D slab was visually positioned the same as in the first study by reference to the spinal cord.

REFERENCE

1. Wang T, Ong CW, Shi J, Srivastava S, Yan B, Cheng CL, Yong WP, Chan SL, Yeoh KG, Iacopetta B, Salto-Tellez M. Sequential expression of putative stem cell markers in gastric carcinogenesis. Br J Cancer. 2011; 105:658-665.

a

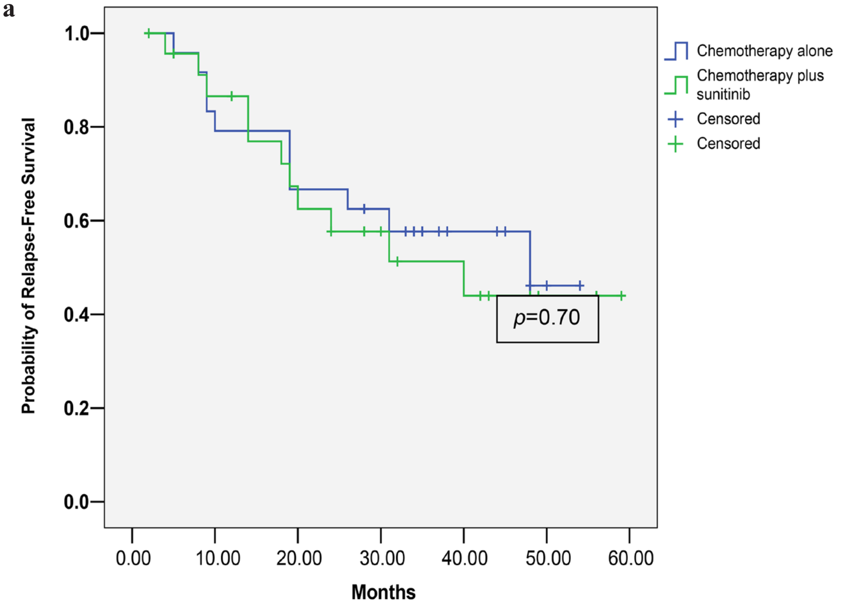

b

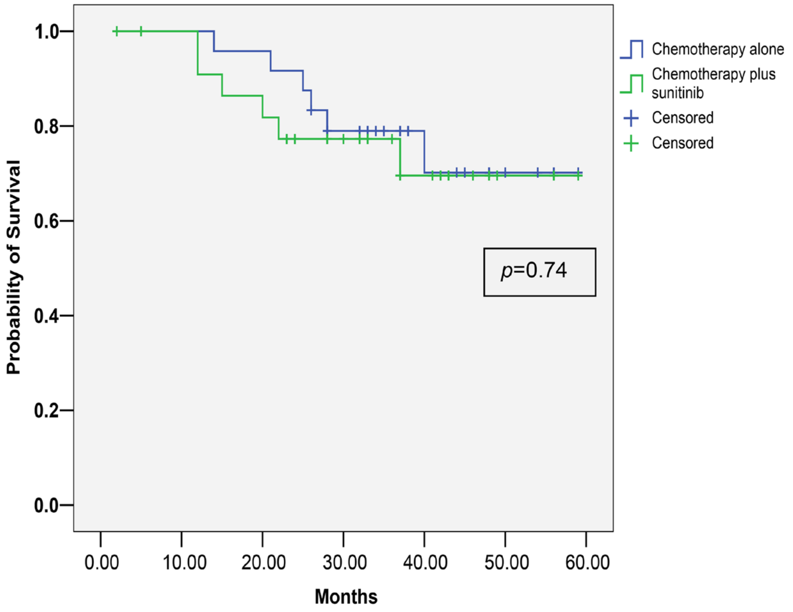

Supplementary Figure S1: a. Kaplan-Meier estimates of progression-free survival by treatment arm, **b.** Kaplan-Meier estimates of overall survival by treatment arm.

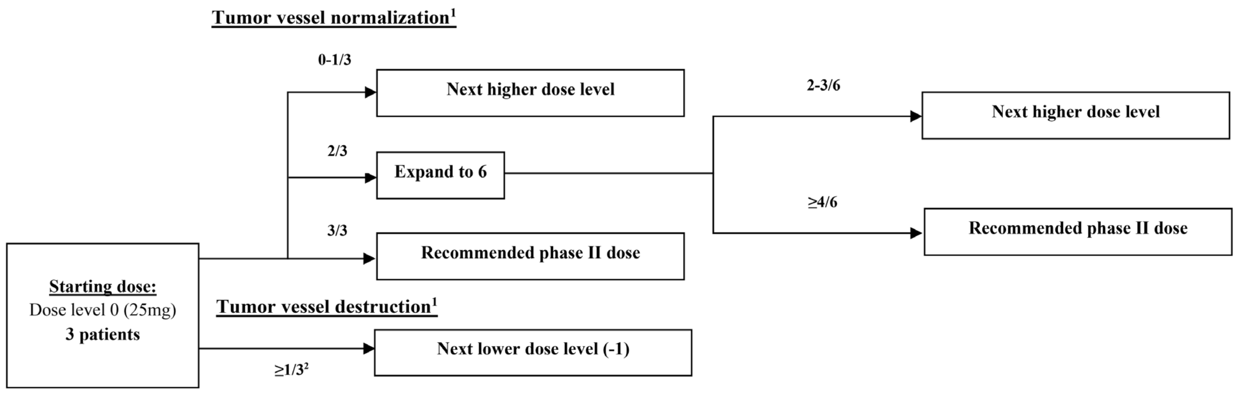

Supplementary Figure S2: Dose-escalation guidelines and dose escalation/de-escalation plan of the phase Ib study.
[1] Tumor vessel normalization/destruction based on immunohistochemical criteria, as described in the main text
[2] Applicable to starting dose level only. At higher dose levels, tumor vessel destruction in ≥2/3 patients is dose-limiting and the next lower dose level would be selected as the recommended phase II dose

Level	Oral sunitinib dose prior to cycle 1 chemotherapy	Oral sunitinib dose prior to each subsequent cycle of chemotherapy
Level -1	12.5 mg daily x 1 week	12.5 mg daily x 1 week
**Level 0**	**25 mg daily x 1 week**	**25 mg daily x 1 week**
Level 1	37.5 mg daily 1 week	37.5 mg daily 1 week
Level 2	25 mg daily x 2 weeks	25 mg daily x 1 weeks
Level 3	37.5 mg daily x 2 weeks	37.5 mg daily x 1 weeks

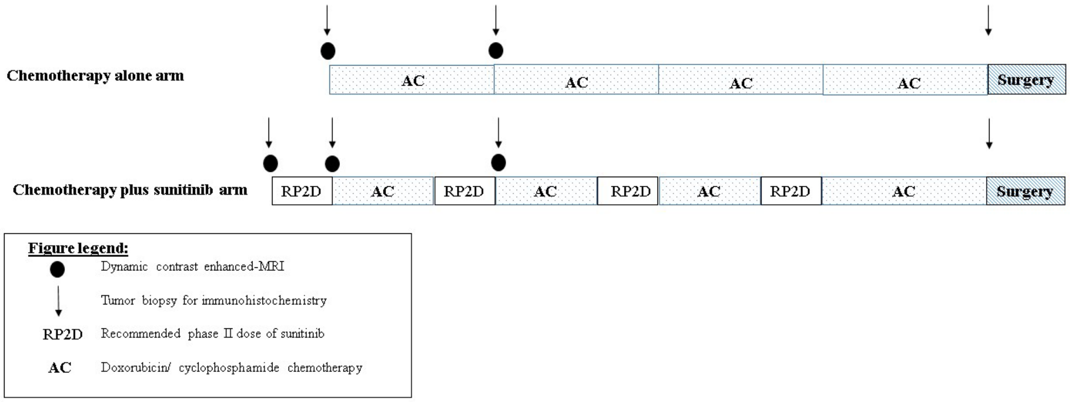

Supplementary Figure S3: Study schema of the randomised phase II study.
